# Semantic feature activation takes time: longer SOA elicits earlier priming effects during reading

**DOI:** 10.1007/s10339-022-01084-3

**Published:** 2022-03-07

**Authors:** Markus J. Hofmann, Mareike A. Kleemann, André Roelke-Wellmann, Christian Vorstius, Ralph Radach

**Affiliations:** General and Biological Psychology, University of Wuppertal, Max-Horkheimer-Str. 20, 42119 Wuppertal, Germany

**Keywords:** Word predictability, word co-occurrence statistics, interactive activation model, Associative-read-out model, Associative and semantic relations

## Abstract

While most previous studies of “semantic” priming confound associative and semantic relations, here we use a simple co-occurrence-based approach to examine “pure” semantic priming, while experimentally controlling for associative relations. We define associative relations by the co-occurrence of words in the sentences of a large text corpus. Contextual-semantic feature overlap, in contrast, is defined by the number of common associates that the prime shares with the target. Then we revisit the spreading activation theory and examine whether a long vs. short time available for semantic feature activation leads to early vs. late viewing time effects on the target words of a sentence reading experiment. We independently manipulate contextual-semantic feature overlap of two primes with one target word in sentences of the form pronoun, verb prime, article, adjective prime and target noun, e. g., "She rides the gray elephant." The results showed that long-SOA (verb-noun) overlap reduces early single and first fixation durations of the target noun, and short-SOA (adjective-noun) overlap reduces late go-past durations. This result pattern can be explained by the spreading activation theory: The semantic features of the prime words need some time to become sufficiently active before they can reliably affect target processing. Therefore, the verb can act on the target noun's early eye-movement measures presented three words later, while the adjective is presented immediately prior to the target—thus a difficult adjective-noun semantic integration leads to a late sentence re-examination of the preceding words.

## Introduction

In early studies of “semantic” priming, associative and semantic relations between the prime and the target were confounded (Lucas 2000; Hutchison 2003; Staub 2015). By using more recent methods based on the co-occurrence of words in large text corpora, associative and semantic priming can be examined independent from each other (Roelke et al. 2018; cf. Mirman et al. 2017; Unger et al. 2020). In the present sentence reading study, we made sure to induce some expectancy of each word by using only content words that are associated to each other (cf. Neely 1977). The aim of the present study was to test a prediction of the spreading activation theory: Collins and Loftus (1975) proposed that the longer a prime concept is processed, the larger is the pre-activation of a semantically similar target. Therefore, a longer stimulus-onset asynchrony (SOA) should lead to larger pre-activation and thus should induce early effects on the target. For a shorter SOA, in contrast, the semantic features of the prime are not sufficiently active to influence target processing early, and thus later effects should be observable. Reaction time studies, however, cannot differentiate between early and late processes. Therefore, we here rely on eye tracking to differentiate between early and late target processing in an ecologically valid reading situation.

In early priming studies, associative relations between words were usually defined by human performance in pre-experimental free association tasks (Lucas 2000; Hutchison 2003). Semantic priming, in contrast, was defined by manually selected word pairs that reflect the idea of a hierarchical knowledge representation taxonomy (Quillian 1967). Associative and semantic priming, however, are typically confounded: As the responses in the free association task mostly also provide a semantic relation to the target, for instance McNamara (2005, p. 86) “challenge[d] anyone to find two highly associated words that are not semantically related in some plausible way” (but cf. Roelke et al. 2018). A similar picture emerges in the sentence reading literature, in which associative, semantic, and syntactic relations are defined by cloze completion probability—thus the resulting empirical predictability can be considered an “all-in” variable (e.g., Brothers and Kuperberg 2021; Ehrlich and Rayner 1981; Staub 2015; Staub et al. 2015). Here we decompose “predictability” by independently manipulating contextual-semantic feature overlap of two primes with one target, while controlling for associative relations and syntactic structure (Hofmann et al. 2018; Roelke and Hofmann 2020).

Methods derived from computational linguistics allow to define more specific types of relations between words. They take a text corpus for training and provide computationally explicit definitions of more specific types of word relations. An early computational method addressing associative priming by direct word contiguity was based on the word co-occurrence probability, divided by the product of the single-word occurrence probabilities (McKoon and Ratcliff 1992). Already Lucas (2000, p. 628) suspected that this “technique could be flawed or of poor resolution,” and indeed recent statistical approaches provide much better predictions for association ratings (Hofmann et al. 2018) or priming (e.g., Unger et al. 2020). Also for sentence reading predictions, there are sophisticated computational approaches that allow to address “low-level” associative priming (McDonald and Shillcock 2003; Smith and Levy 2013). To address high-level semantic knowledge, in contrast, latent semantic analysis (LSA) is probably the best-known computational approach in psychology. Landauer and Dumais (1997) computed latent semantic dimensions that determine the co-occurrence of words in large text corpora. The probably only eye-tracking study that contrasted word viewing times due to associative vs. semantic relations was conducted by Wang et al. (2010; see also Luke and Christianson (2016)). They found that the associative relation to the preceding word affects early viewing time measures (McDonald and Shillcock 2003), while a semantic relation to the preceding word affects late viewing time measures (see also Frank and Willems, 2017, for a similar approach to brain-electric and neuroimaging data).

**Fig. 1:**
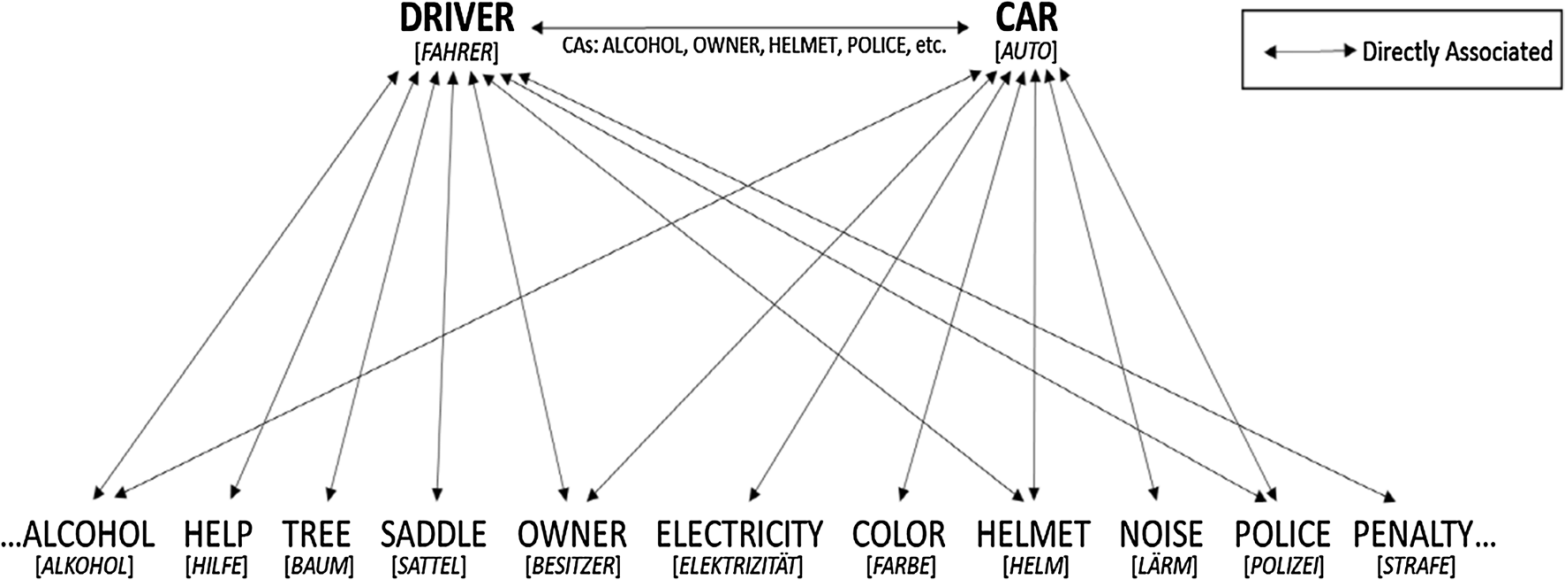
A simple approach to disentangle (associative) contiguity and contextual-semantic feature overlap of two words. Driver and car often co-occur in the same sentence and therefore are associated, but they also provide many common associates, e.g., alcohol, owner and helmet (cf. e.g., Roelke et al. 2018)

Roelke and colleagues (2018) demonstrated that associative, as well as semantic relations can be comprehensively captured by a single computational approach. They defined two words as associated, if they co-occur significantly more often together in the sentences of a large text corpus (*P* < 0.01; cf. McDonald and Shillcock 2003)—otherwise the words are not associated (cf. Unger et al. 2020). If the words are directly associated, however, continuous association strength is defined by the log-transformed *χ*^2^ value of this simple log likelihood test (Dunning 1993; cf. Hofmann et al. 2011). Contextual-semantic feature overlap, in contrast, was defined by the number of significant common associates, the prime and the target share (Hofmann et al. 2018; cf. Landauer and Dumais 1997; Rapp 2002). For instance, the prime “driver” and the target “car” were directly associated, but they also provided common contextual features such as “alcohol” or “helmet,” while other features were associated only with one of the stimuli, e.g., saddle and tree were associated with “driver,” but not with “car” (see Fig. 1). By computing these measures for a large list of words, Roelke et al. (2018) were also able to answer McNamara’s (2005, p. 86) challenge to find highly associated words that are not semantically related, such as “cold—hunger” or “devil—detail.” In their primed lexical decision study, Roelke et al. (2018) then found “pure” associative priming effects at a short (200 ms) and at a long (1000 ms) SOA, while “pure” semantic priming effects were constrained to a short SOA. This RT pattern is similarly observed in the classic priming literature (Ferrand and New 2003; Hutchison 2003; Lucas 2000). The lack of a pure semantic priming effect at a long SOA can be explained by the interplay of associative and semantic processes: Facilitation is observed when an (associative) expectancy is met, otherwise strong semantic competitors can also lead to inhibition (Neely 1977). Though RT data did not indicate semantic effects at a long SOA, error data indicated some semantic priming effect, when an associative relation is likewise present (Roelke et al. 2018). To consistently induce an expectancy for all targets, we here made sure that all primes are associated with the targets. Moreover, since lexical decision task RTs can hardly differentiate between lexical, decision-related and postlexical checking processes (Balota et al. 1992; Balota and Chumbley 1984), we here examine differential viewing time parameters during natural reading.

Though Collins and Loftus (1975) seminal work did not differentiate between associative and semantic priming (Lucas 2000; Hutchison 2003), their theory allows for a dynamic perspective on semantic integration. They proposed that when a prime word is presented, activation can spread from the prime node toward the nodes of semantically similar target words (Hofmann et al. 2011; Hofmann & Jacobs 2014; McClelland and Rumelhart 1981). When such a similar target is presented later, semantic processing is facilitated, which leads to faster response times as opposed to non-similar targets (e.g., Lucas 2000). Concerning the dynamics of semantic processing, they suggested that “[t]he longer a [prime] concept is continuously processed […], the longer activation is released from the node of the concept” (Collins and Loftus 1975, p. 411). As a consequence, the pre-activation of a semantically similar target should be larger, when a long time passes after prime presentation, before the target is presented. Therefore, we expected early “pure” semantic priming effects at a relatively long SOA. At a short SOA, in contrast, the overlapping semantic features of the prime and the target may not yet be sufficiently active to elicit an early effect. Therefore, we expected late effects of semantic integration when a short time passes between prime and target onset.

Though Roelke et al.’s (2018) primed lexical decision experiment could not differentiate between early and late cognitive target processing, they obtained a result pattern that is consistent with Collins and Loftus (1975) idea of larger pre-activation at a long SOA: They found that associative priming effects are greater at a longer SOA. In the present study, we experimentally control for associative relations, and test whether this result pattern can also be observed for semantic priming. To induce a certain amount of expectancy for the target (Balota et al. 1992; Neely 1977), we made sure that the two primes are associated with the target, with a low and experimentally controlled association strength (Hofmann et al. 2011). We presented sentences of the form pronoun, *verb*, article, *adjective* and noun, followed by series of further words, for instance, “She *rides* the *gray* elephant on one of her many exploratory voyages.” We experimentally manipulated contextual-semantic feature overlap of the *verb* and the *adjective* primes to the target noun by simply counting the number of common associates (Hofmann et al. 2018; Roelke et al. 2018). As a longer time passes between the verb and the target, this experimental manipulation effectively reflects a long SOA. Between the adjective prime and the target noun, in contrast, there is less time for associative spreading, effectively reflecting a short SOA. Rather than using only the behavioral end product as reflected in reaction times, we here examined fixation durations using eye tracking, which allows to differentiate early from late cognitive processes during target processing. If associative spreading activation elicited by the prime indeed takes some time before it can reliably affect target processing (Collins and Loftus 1975), early effects of spreading activation can be expected at a long SOA, while short-SOA priming may elicit later effects in eye movements.

An early eye-tracking effect is apparent, when only one gaze is sufficient for recognizing the target word (e.g., Inhoff and Radach 1998; Rayner 1998). In this case, the single fixation duration informs about the duration of rapidly successful lexical access. Another parameter indexing early processing is the first fixation duration, when further including words that receive more than one fixation during first-pass reading. When adding the duration of further fixations in the (first-pass) gaze duration, initial lexical access should be finished (see Radach and Kennedy 2013). While the total viewing duration includes target re-reading after visiting experimentally non-controlled words to the right of the target, this late parameter is only reported but not discussed. Rather, we focused on the go-past duration (e.g., Schotter 2013)—a measure that includes leftward regressions to account for late semantic integration effort.

There is some evidence in the eye-tracking literature that already supports our hypothesis. Carroll and Slowiaczek (1986) found semantic priming in eye movements at a short (488 ms) and a long SOA (1247 ms), though their early study relied on the mean fixation duration of all fixations of a word during first-pass reading. Because this measure confounds early and late fixation duration measures (Inhoff and Radach 1998), they were not able to differentiate between early and late effects on eye movements. Traxler et al. (2000, experiment 1) examined semantic priming at a relatively short SOA by presenting sentences like “The lumberjack *carried* the axe early in the morning.” When replacing the verb prime by schema-inconsistent verbs like “chopped,” they observed a slower total viewing duration, but no effects of first fixation or first-pass gaze duration, supporting the hypothesis that a relatively short prime-target SOA elicits late semantic priming effects (cf. Wang et al. 2010). We expect to constrain such a late effect to pure semantic priming, while making sure that all prime and target words are associated and keeping association strength constant (cf. Roelke et al. 2018). As we did not control for the words to the right of the target, we expect such short-SOA priming effects in the go-past duration. For long-SOA priming, in contrast, Collins and Loftus (1975) framework predicts an early effect on eye movements, because the prime node had more time to excite pre-activation in the target node and its semantic features. Therefore, we expect these early effects in the single and first fixation duration.

## Method

### Participants

Thirty-two German native speakers with normal or corrected-to normal vision and without language disorders participated in the study for cash or course credits. Two participants were excluded, because they deviated more than 2 SD (SD = 3.76) from the mean error score (*M* = 6.38; range = 0 to 17) in the comprehension test (see Procedure below). The remaining 30 subjects had a mean age of 23.60 years (SD = 5.86, range = 19 to 44, 21 female).

### Materials

Stimuli consisted of 160 German pronoun-verb-article-adjective-noun sentences, continued with three closed-class words (articles, prepositions, conjunctions or pronouns) and 1–6 additional words. Forty filler sentences contained no open-class words from the experimental stimuli and did not follow any syntactic construction rule. Sentences consisted of 69–72 characters and 9–14 words. Verb-noun and adjective-noun contextual-semantic feature overlap was manipulated by the number of common associates (Hofmann et al. 2018). All computations were based on the lemmas accumulated in the German corpus of the Leipzig Wortschatz Project^1^ (70 million sentences, 1.1 billion words; Goldhahn et al. 2012). We used the 1000 words with the largest association strength (Dunning 1993; Hofmann et al. 2011) and counted the number of common associates of each prime-target pair. To constrain the common associates to words relatively diagnostic for a particular meaning, we excluded the 100 most frequent words (Griffiths et al. 2007; Hofmann et al. 2018). With the second experimental factor of verb vs. adjective reflecting a long vs. short SOA, this resulted in four experimental conditions each containing 40 sentences (Table 1).

**Table 1:** Example sentences for the experimental conditions

	CA with target		Example
Prime:	Verb	Adjective	
SOA:	Long	Short	
HH	High	High	Sie *reitet* den *grauen* Elefanten auf einer ihrer vielen Forschungsreisen. (She *rides* the *gray* elephant on one of her many exploratory voyages.)
HL	High	Low	Er *zeigt* das *amtliche* Muster seinen in einem Büroraum wartenden Kollegen. (He *shows* the *official* sample to his colleagues waiting in an office room.)
LH	Low	High	Sie *testet* den *flinken* Frosch mit einer von ihr entwickelten Messmethode. (She *tests* the *swift* frog with a measuring method developed by her)
LL	Low	Low	Er *erwarb* das *klapprige* Gefährt mit einem seiner ungedeckten Schecks. (He *acquired* the *shaky* vehicle with one of his uncovered checks.)

CA – Number of common associates of *prime* and target words. High: CA > 60; Low: CA < 15 CA

We controlled Leipzig word frequency classes relating the frequency of each word to the frequency of the most frequent German word: “der” [the] is 2^class^ more frequent than the given word (Goldhahn et al. 2012). Length, frequency, and number of orthographic neighbors of the prime and target words, prime-target association strength, association strength and common associates between the primes, as well as length and frequency of the closed-class words after the target were experimentally controlled (all *F*s < 1, see Table 2 and Appendix A). Half of the experimental trials in each stimulus category included a comma after the target to obtain syntactic variety.

**Table 2:** Mean values (*SD* in parentheses) of manipulated and controlled variables

	HH	HL	LH	LL
CA Verb-noun	78.93 (15.82)	78.28 (18.47)	10.88 (3.33)	10.68 (3.05)
CA Adjective-noun	86.05 (23.57)	10.38 (3.61)	85.08 (25.72)	11.18 (3.86)
AS Verb-noun	1.27 (0.32)	1.26 (0.32)	1.19 (0.31)	1.23 (0.26)
AS Adjective-noun	1.24 (0.36)	1.19 (0.27)	1.26 (0.33)	1.16 (0.26)
*Target noun*				
Length	6.10 (1.43)	6.15 (1.33)	6.23 (1.21)	5.98 (1.46)
Frequency	11.38 (1.69)	10.90 (1.84)	11.20 (1.77)	11.53 (2.72)
ON	1.88 (3.20)	1.68 (1.95)	1.30 (1.96)	1.53 (2.16)
*Verb*				
Length	7.03 (0.92)	6.88 (1.07)	7.18 (0.90)	7.03 (0.86)
Frequency	12.33 (1.87)	12.65 (2.39)	12.85 (3.85)	12.60 (3.69)
ON	2.58 (2.95)	2.33 (2.76)	1.85 (2.15)	2.20 (2.40)
*Adjective*				
Length	6.45 (1.11)	6.53 (1.18)	6.25 (1.41)	6.38 (1.37)
Frequency	13.65 (1.31)	13.25 (4.06)	12.98 (2.03)	13.23 (3.13)
ON	0.55 (0.81)	0.73 (1.11)	0.75 (1.32)	0.90 (1.17)

CA = Number of common associates, AS = Association strength; ON = Number of orthographic neighbors

### Procedure

Participants were instructed to read silently at their normal pace to be able to respond to comprehension questions. Eye movements were recorded by an EyeLink1000® (2000 Hz, SR Research, Toronto, Canada). Participants used a chin- and forehead-rest to minimize head movements. Three-point calibration was performed at the beginning of the experiment, after every block and after each comprehension question. The experiment started with 12 practice trials. Each trial started with a fixation point presented approximately one letter to the left of the beginning of the first word, simultaneously serving as drift check. Deviations greater 0.33° triggered an additional calibration. Sentences were displayed as single lines in black font (Courier New, 18 pt) on a light-gray background, vertically centered on a 24-inch flat panel monitor (1680 × 1050 pixel, 120 Hz; viewing distance: 68.75 cm). A letter corresponded to a visual angle of 0.33°. 1000 ms blank screens were presented after participants initiated the next trial by button press. After each practice trial and after a randomly selected third of the main experimental trials (67 questions), we presented comprehension questions, which were answered orally. The 200 sentences were pseudorandomized in two lists with no more than two sentences of the same experimental condition to appear consecutively. We split these lists into two blocks, making sure that the first and second block had approximately the same number of sentences of each category, also balancing list and block order across participants.

### Analyses

Right-eye fixations on critical target words were analyzed if both primes were fixated before. We removed fixations < 70 ms and > 800 ms for single and first fixation duration, > 1000 ms for gaze duration, and > 1500 ms for go-past and total viewing duration. Inferential statistics were based on linear mixed models (LMMs) with maximum likelihood estimation (*lme4* and *lmerTest* packages in R). Fixed effects were the number of common associates of verb and adjective to the target word (low vs. high) and their interaction, using successive differences coding (− 0.5 vs. 0.5; contr.sdif, *MASS* package). LMMs started with maximum random structure including random slopes for both effects (Barr et al. 2013). We simplified LMMs by removing random slopes for interactions and main effects that led to singular matrices or failure to converge (cf. Baayen 2008). Final models contained random item and subject intercepts (Bates et al. 2015). We removed trials in which residuals deviated more than 2.5 SD from mean (see Table 4 for remaining trials). Kolmogorov–Smirnov tests for all final models indicated no significant deviance from normality (*P*s > 0.05), except for gaze and total viewing duration analyses (*Ps* = 0.007). Eye-movement data were log-transformed for inferential statistics as displayed in Table 4, but Table 3 reports the non-transformed values. To test whether the effects at one SOA are significantly stronger than the effects at the other SOA, we build LMMs containing only the verb or the adjective fixed factor and compared them using log likelihood tests (R anova function).

**Table 3:** Means (*SE*) of the target noun for the different eye-movement parameters

	HH	HL	LH	LL
SFD	235 (3)	233 (3)	240 (3)	244 (3)
FFD	234 (3)	232 (3)	240 (3)	244 (3)
GD	262 (4)	257 (4)	265 (4)	280 (5)
TVD	419 (7)	374 (7)	355 (8)	358 (9)
GPD	601 (11)	633 (15)	637 (13)	710 (14)

SFD = Single fixation duration; FFD = First fixation duration; GD = Gaze duration; TVD = Total viewing duration GPD = Go-past duration

## Results

An average time of 1152 ms (*SE* = 8) passed between the verb prime and target noun fixation onsets in the long-SOA conditions. Fixation onsets of adjectives and nouns differed by a *M* = 515 ms (*SE* = 6) in the short-SOA conditions.

In the single fixation duration analyses, we found early verb priming effects of long-SOA contextual-semantic feature overlap as defined by the number of common associates with the target (*P* = 0.035; Tables 3 and 4). Neither the adjective prime nor the interaction revealed a reliable effect (*Ps* > 0.1). An LMM containing only the verb performed significantly better than an LMM containing only the adjective factor, suggesting that successful word recognition at a single glance is more likely driven by long-SOA priming (*χ*^*2*^ = 4.389; df = 0; *P* < 0.01).

**Table 4:** Results of the LMM analyses (* P < 0.05)

	N rows	Intercept		CA verb			CA adjective			CA Verb * CA adjective			Random intercept σ^2^		Residual σ^2^
		B	SE	B	SE	T	B	SE	T	B	SE	T	Item	Subject	
SFD	3179	5.41	0.03	− 0.03	0.02	− 2.13 *	0	0.02	0.08	0.03	0.03	1.04	0.01	0.03	0.07
FFD	3702	5.4	0.03	− 0.04	0.01	− 2.54 *	0	0.01	− 0.29	0.02	0.03	0.81	0.01	0.03	0.07
GD	3716	5.48	0.03	− 0.04	0.02	− 1.92	− 0.02	0.02	− 0.7	0.07	0.05	1.6	0.02	0.03	0.10
TVD	3722	5.77	0.05	− 0.07	0.03	− 1.95	− 0.04	0.03	− 1.2	0.11	0.07	1.61	0.04	0.06	0.17
GPD	1134	6.24	0.03	− 0.05	0.03	− 1.67	− 0.06	0.03	− 1.98 *	0.05	0.07	0.83	0.02	0.03	0.12

For the first fixation duration, we found a reliable effect of verb-noun semantic feature overlap (*P* = 0.012). Neither the effect of the adjective prime nor the interaction was significant. The LMM containing only the verb factor performed significantly better than the adjective LMM (*χ*^*2*^ = 6.21; df = 0; *P* < 0.001). For the gaze duration analysis, we obtained no reliable effects, though the verb effect marginally failed to reach significance (*P* = 0.057). The analysis of total viewing duration revealed no reliable effects, though the verb factor marginally missed significance (*P* = 0.053). In sum, these results suggest that verb priming is more likely to affect earlier viewing time parameters.

In the analysis of the go-past duration, the verb effect marginally missed significance (*P* = 0.098, Table 4). There was no significant interaction, but a reliable effect for the adjective prime (*P* = 0.049). The adjective LMM better fitted the data than the verb model (*χ*^*2*^ = 1.1034; df = 0; *P* < 0.001). This late eye-movement parameter is primarily driven by semantic priming at a short SOA.

## Discussion

In the classic priming literature, semantic priming is typically confounded with associative priming (Lucas 2000; Hutchison 2003). To induce a certain amount of expectancy (Neely 1977), we used simple word co-occurrence to make sure that all content words of our stimulus sentences are associated. Then we defined the contextual-semantic features by the common associates and examined the prediction of the spreading activation theory that semantic feature activation takes time during natural reading. We found that pure semantic priming at a long SOA (*M* = 1152 ms) elicits early effects at the level of the single and first fixation duration. At a short SOA (*M* = 515 ms), in contrast, we found late effects in the go-past duration only.

The present results are quite comparable to the results of Roelke et al. (2018), who found RT effects of semantic priming at a short, but not at a long SOA. While short-SOA priming elicited an average go-past duration facilitation of 47 ms (SE = 15), long-SOA priming elicited a relatively small facilitation of 7/8 ms in single and first fixation durations, respectively (SEs = 3). Moreover, it should be noted that Roelke et al. (2018) found a main effect of pure semantic priming in their error data, while the interaction indicated that this effect results from the associatively related word pairs. In the present study, we made sure that there is an association between the primes and the target. Therefore, we conclude in line with Roelke et al. (2018) that pure semantic priming effects occur at a long SOA, when an associative relation is likewise present.

The result pattern of long-SOA priming affecting early and short-SOA priming affecting late processes can be explained by the time available for associative spreading (cf. Collins and Loftus 1975): When a prime is presented, its semantic features become active, but they need time to become sufficiently active to allow for a rapid activation of the target. The stronger the activation of the semantic features of the prime, the more immediate will be the interaction with the overlapping semantic features of the target. With a long SOA, the features of the verb prime had sufficient time to become active, leading to early single and first fixation duration effects. At a short SOA, in contrast, the adjective’s semantic features did not have enough time to become sufficiently active to elicit an early effect. Therefore, they influence later prime-target semantic integration, as reflected in the go-past duration. After first-pass reading has been finished, a sufficient period of time has elapsed for the semantic features of the adjective and the noun to become active: When there is a high semantic feature overlap, the adjective and the noun can be semantically integrated with ease, but when there is a low overlap, semantic integration is more likely to fail. When assuming a semantic layer feeding activation to a lexical layer (e.g., Hofmann and Jacobs 2014; McNamara 2005, p. 41), lower-level saliency of the preceding letters and words is increased (see Reilly and Radach, 2006, Fig. 2). Therefore, the preceding sentence context gains attention and is selected as the target location of further eye movements. In other words, postlexical semantic integration mechanisms interact with attentional saliency and lead to a re-examination of the preceding sentence (cf. Balota et al. 1992; Reilly and Radach, 2006; Snell et al. 2018).

Most of the previous definitions of semantic relations between words in sentences confound the semantic expectations generated by several words. For instance, the classic approach to semantics during sentence processing relies on pre-experimental samples in cloze completion tasks (e.g., Brothers and Kuperberg 2021; Ehrlich and Rayner 1981; Taylor 1953). The resulting empirical predictability can be considered an “all-in” variable that confounds syntactic, associative and semantic expectations about upcoming words (Staub et al. 2015). In the present study, we kept syntax and associative relations between words constant, thus examining “pure” effects of contextual-semantic feature overlap. We think that this is an important step toward de-composing the “all-in” variable of empirical predictability into more specific sub-processes (cf. Staub 2015).

To our knowledge, there is only a single eye-tracking study that took into account an associative and a semantic predictor variable. Wang et al. (2010) showed that a direct association to the preceding word elicits an early effect in the first fixation duration, while semantic similarity to the last content word engaged a late eye-movement effect in the total viewing duration. Our results corroborate their conclusion that the semantic integration of the last and the present content word occurs late (cf. Traxler et al. 2000). The present study, however, is the first to show that pure semantic priming elicits early effects during target processing, when there is more time for the prime to pre-activate the semantic features of the target and potentially also the target itself (cf. Radach and Hofmann 2016, Fig. 2).

While most of the eye-tracking studies on sentence reading examine computationally defined semantic or associative measures in regression analyses (e.g., Boston et al. 2008; Demberg and Keller 2008; Frank and Bod 2011; Luke and Christianson 2016; Smith and Levy 2013), we believe that manipulating and controlling for differential computational parameters will be essential for obtaining a reliable and “deep explanation (…) of higher-level linguistic processing” that is still lacking in present models of eye-movement control (Reichle et al. 2003, p. 450; cf. Engbert et al. 2005). Particularly for eye-tracking research, analytical problems resulting from confounded manipulations and multivariate covariates are critical in regression analyses (Matuschek and Kliegl 2018; Rayner et al. 2007; cf. Kliegl et al. 2006). To our knowledge, McDonald and Shillcock (2003) generated the only stimulus sentences, in which a computational measure of associative relations was manipulated, while experimentally controlling for confounding variables such as word frequency and length. In the present study, we additionally controlled associative relations to exclusively examine semantic priming.

Though we think that experimental approaches are essential for obtaining consistent and reliable conclusions about semantic priming, the experimental approach likewise leads to a lack of generality across different types of word classes (see e.g., Hofmann & Jacobs 2014). In the present study, we experimentally controlled for the syntactic structure of our stimulus sentences. Therefore, a first important limitation of the present study is that our conclusions are constrained to long-SOA verb-noun integration and short-SOA adjective-noun integration. Within the theoretical framework of spreading activation, such a confound is expectable, because the use of particular word classes is constrained by syntax (Collins and Loftus 1975, p. 408f) and “syntax and semantics are surely interwoven” (Quillian 1967, p. 428). When assuming a strict separation of syntax and semantics, however, an alternative post-hoc explanation may account for the present findings. This alternative theoretical perspective proposes that our stimulus sentences consisted of pronoun, *predicate-prime*, article, *adjective prime* and target-object. Because predicate-object integration might be more central for understanding the gist of the sentences, it is possible that not the time between prime and target, but this centrality was critical for preferential processing at an early “stage” of processing (cf. Traxler et al. 2000). Adjective-object semantic feature overlap in contrast might induce a more peripheral type of semantic integration at a lower level of the syntactic hierarchy (e.g., Prior and Bentin 2006; Zhang et al. 2011). This more peripheral type of semantic integration may be the actual cause for local re-checking of the preceding sentence, as reflected in the go-past duration. These ERP studies, however, did not show that central vs. peripheral semantic integration affects differential time windows. Nevertheless we suggest that future studies should manipulate SOA together with central vs. peripheral semantic integration to further corroborate that the SOA determines early vs. late target processing. To examine whether a short SOA is critical for the late eye-movement effects, for instance, the article and adjective could be eliminated from the present stimuli (“She rides elephants”). When early effects are apparent at the level of single and first fixation duration, preferential processing of central semantic integration would be a more appropriate explanation. When the time required for semantic feature activation is the appropriate explanation, however, we would expect late effects that are still consistent with the spreading activation theory.

If the first explanation is supported by empirical data, future computationally concrete tests of Collins and Loftus (1975) seminal ideas should be based on syntax-specific computations of semantic similarity. In fact, there is a computational linguistics approach that counts only the common associates of syntactically combinable word classes to compute semantic feature overlap at the level of memory consolidation (Biemann and Riedl 2013,2). This leads to the second most notable limitation of the present study that word 2vec models usually deliver more accurate estimates of semantic and/or syntactic similarity than simple common associates as a proxy of symbolic semantic features (Hofmann et al. 2018; Mandera et al. 2017; Mikolov et al. 2013). Nevertheless, we think that simple symbolic computational estimates of semantic similarity are worth to be investigated, because they deliver an epistemically more transparent explanation than subsymbolic approaches (see Hofmann and Jacobs 2014).

## Appendix

See Table

**Table 5:** Mean values (*SD* in parentheses) and *F*-scores of the controlled variables

	HH	HL	LH	LL	F
AS verb-adjective	0.00 (0.00)	0.02 (0.13)	0.00 (0.00)	0.02 (0.14)	0.68
CA verb-adjective	29.50 (10.44)	25.93 (18.54)	28.28 (14.73)	27.40 (22.66)	0.31
Closed-class word 1					
Length	3.25 (1.26)	3.38 (1.43)	3.28 (1.22)	3.38 (1.39)	0.10
Frequency	3.10 (2.41)	3.65 (2.82)	2.98 (2.25)	3.08 (2.39)	0.61
Closed-class word 2					
Length	3.38 (1.15)	3.40 (1.15)	3.30 (1.04)	3.38 (1.08)	0.06
Frequency	2.68 (1.59)	2.55 (1.41)	2.58 (1.41)	2.25 (1.46)	0.62
Closed-class word 3					
Length	3.35 (0.89)	3.43 (0.75)	3.53 (1.09)	3.40 (1.13)	0.23
Frequency	2.63 (1.90)	2.50 (1.54)	2.88 (1.64)	2.63 (1.55)	0.36

AS = Direct associative strength; CA = Number of common associates; HH = high number of CA between both primes and noun; HL = High number of CA between verb and noun and low number of CA between adjective and noun; LH = Low number of CA between verb and noun and high number of CA between adjective and noun: LL = Low number of CA between both primes and target

5.

## Footnotes

1 http://www.corpora.uni-leipzig.de/en?corpusId=deu_newscrawl-public_2018.

2 http://ltmaggie.informatik.uni-hamburg.de/jobimviz/.
